# Supplementary material for: Context information supports serial dependence of multiple visual objects across memory episodes
Source: Nat Commun. 2020 Apr 22;11:1932. doi: 10.1038/s41467-020-15874-w (PMC7176712; doi:10.1038/s41467-020-15874-w)
Supplement: Supplementary file 1 — Supplementary Information [file 41467_2020_15874_MOESM1_ESM.pdf]

Supplementary Information

Context information supports serial dependence of multiple visual objects across memory episodes

Fischer et al. (2020)

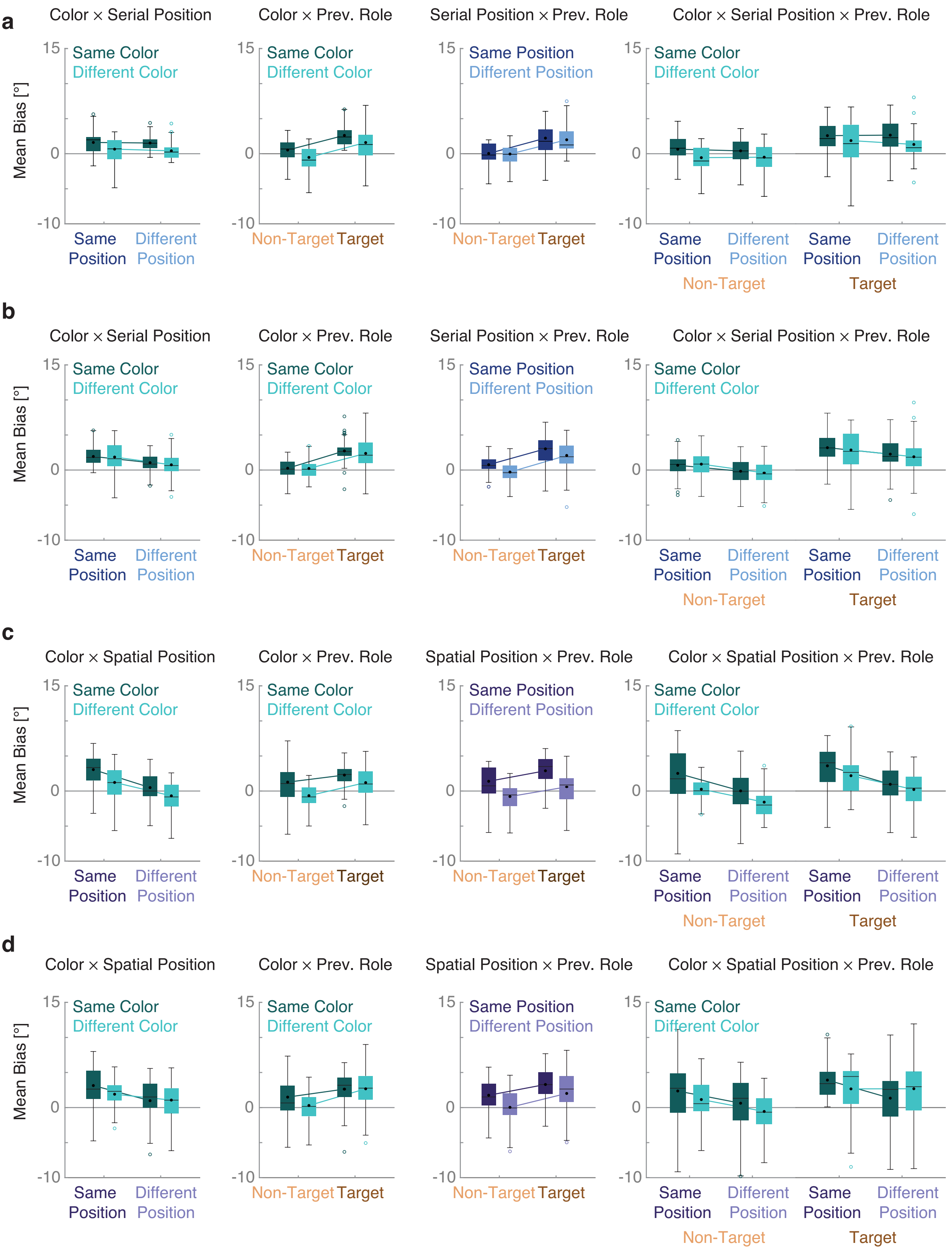

Supplementary Figure 1. Interaction results of Experiments 1-4. Panels in **a**) to **d**) depict the group mean bias aggregated across two levels of a single factor (previous role, serial/spatial position and color) or plotted separately for each condition to illustrate the absence of 2-way (Panel 1 to 3 from left to right) or 3-way-interactions (rightmost panel), respectively. Panels in **a**) and **b**) show results for Experiments 1 and 2 and in **c**) and **d**) for Experiments 3 and 4, respectively. The y-axis indicates the mean response bias in degrees with a positive bias indicating attraction toward a previous item. Box-plots depict the area between the first and the third quartile (box edges) and the median (box center, solid line). Whiskers depict maximally 1.5 times the Interquartile Range (IQR), with the endpoint of the whisker adjusted to the minimum/maximum data point, if it falls below 1.5*IQR. Data points that fall outside of 1.5*IQR are depicted as colored circles. Additionally, the mean is depicted as a solid black circle within the boxes (four experiments with independent samples; Exp. 1: n=20, Exp. 2: n=49, Exp. 3: n=20, Exp. 4: n=20). This figure illustrates the relationship between context features and the previous role of an item for all four experiments (see Supplementary Tables 1-4 for statistical results). Source data are provided as a Source Data file.

Supplementary Table 1. ANOVA results for Experiment 1.

Factor	*df*	*F*	*p-Value*	BF_{incl}	η_p^2
Color	1,19	13.821	**.001**	**106.896**	0.421
Serial Position	1,19	0.892	.357	0.182	0.045
Previous Role	1,19	19.949	**<.001**	**$3.829*10^9$**	0.512
Color × Serial Position	1,19	0.176	.679	0.250	0.009
Color × Previous Role	1,19	0.005	.944	0.227	0.000
Serial Position × Previous Role	1,19	0.100	.755	0.246	0.005
Color × Serial Position × Previous Role	1,19	0.296	.312	0.473	0.054

Bold text highlights p-values < .05 and their accompanying Bayes Factors (BF_{incl}) which indicate the contribution of a single factor or interaction. None of the interactions was significant (lowest *p = .312*). For all 2-way interactions, the BF_{incl} were below 1/3, which indicates that the data were more likely to occur under a model without those interactions. The 3-way interaction Color × Serial Position × Previous Role had a BF_{incl} of 0.473 (*p = .312*), which indicates inconclusive evidence. Source data are provided as a Source Data file.

Supplementary Table 2. ANOVA results for Experiment 2.

Factor	*df*	*F*	*p-Value*	BF_{incl}	η_p^2
Color	1,48	0.768	.385	0.204	0.016
Serial Position	1,48	25.941	**<.001**	**$1.442*10^5$**	0.351
Previous Role	1,48	101.993	**<.001**	**$7.362*10^{27}$**	0.680
Color × Serial Position	1,48	0.818	.370	0.165	0.017
Color × Previous Role	1,48	1.024	.317	0.209	0.021
Serial Position × Previous Role	1,48	0.085	.772	0.163	0.002
Color × Serial Position × Previous Role	1,48	0.296	.589	0.359	0.006

Bold text highlights p-values < .05 and their accompanying Bayes Factors (BF_{incl}) which indicate the contribution of a single factor or interaction. None of the computed interactions reached significance (lowest *p = .317*). For all 2-way interactions, the BF_{incl} were below 1/3, which indicates that the data were more likely to occur under a model without those interactions. The 3-way interaction Color × Serial Position × Previous Role had a BF_{incl} of 0.359 (*p = .589*), which indicates inconclusive evidence. Source data are provided as a Source Data file.

Supplementary Table 3. ANOVA results for Experiment 3.

Factor	*df*	*F*	*p-Value*	BF_{incl}	η_p^2
Color	1,19	10.853	**.004**	**139.569**	0.364
Spatial Position	1,19	37.623	**<.001**	**$3.082*10^5$**	0.664
Previous Role	1,19	10.863	**.004**	**78.900**	0.364
Color × Spatial Position	1,19	0.866	.364	0.313	0.044
Color × Previous Role	1,19	1.288	.270	0.426	0.063
Spatial Position × Previous Role	1,19	0.019	.891	0.226	0.001
Color × Spatial Position × Previous Role	1,19	<0.001	.995	0.276	0.000

Bold text highlights p-values < .05 and their accompanying Bayes Factors (BF_{incl}) which indicate the contribution of a single factor or interaction. None of the computed interactions was significant (lowest *p = .270*). For the 2-way interactions Color × Spatial Position as well as Spatial Position × Previous Role, the BF_{incl} was below 1/3, which indicates that the data were more likely to occur under a model without those interactions, rather than a model including them. For the 2-way interaction Color × Previous Role, the BF_{incl} was 0.426 (*p = .270*), which indicates inconclusive evidence. The 3-way interaction Color × Serial Position × Previous Role had a BF_{incl} of 0.276 (*p = .995*), indicating that the data was more likely to occur under a model without this interaction. Source data are provided as a Source Data file.

Supplementary Table 4. ANOVA results for Experiment 4.

Factor	*df*	*F*	*p-Value*	BF_{incl}	η_p^2
Color	1,19	1.316	.266	0.329	0.065
Spatial Position	1,19	6.285	**.021**	**4.701**	0.249
Previous Role	1,19	5.790	**.026**	**15.191**	0.234
Color × Spatial Position	1,19	0.992	.332	0.352	0.050
Color × Previous Role	1,19	2.009	.173	0.338	0.096
Spatial Position × Previous Role	1,19	0.197	.662	0.228	0.010
Color × Spatial Position × Previous Role	1,19	1.392	.253	0.518	0.068

Bold text highlights p-values < .05 and their accompanying Bayes Factors (BF_{incl}) which indicate the contribution of a single factor or interaction. None of the interactions was significant (lowest p = .173). For the 2-way interaction Spatial Position × Previous Role, the BF_{incl} was below 1/3, which indicates that the data was more likely to occur under a model without those interactions, rather than a model including them. For the 2-way interactions Color × Spatial Position and Color × Previous Role, the BF_{incl} were just above 1/3, with BF_{incl} of 0.352 ($p = .332$) and 0.338 ($p = .173$), respectively, which indicates inconclusive evidence. The same was true for the 3-way interaction Color × Spatial Position × Role (BF_{incl} = 0.518, $p = .253$). Source data are provided as a Source Data file.

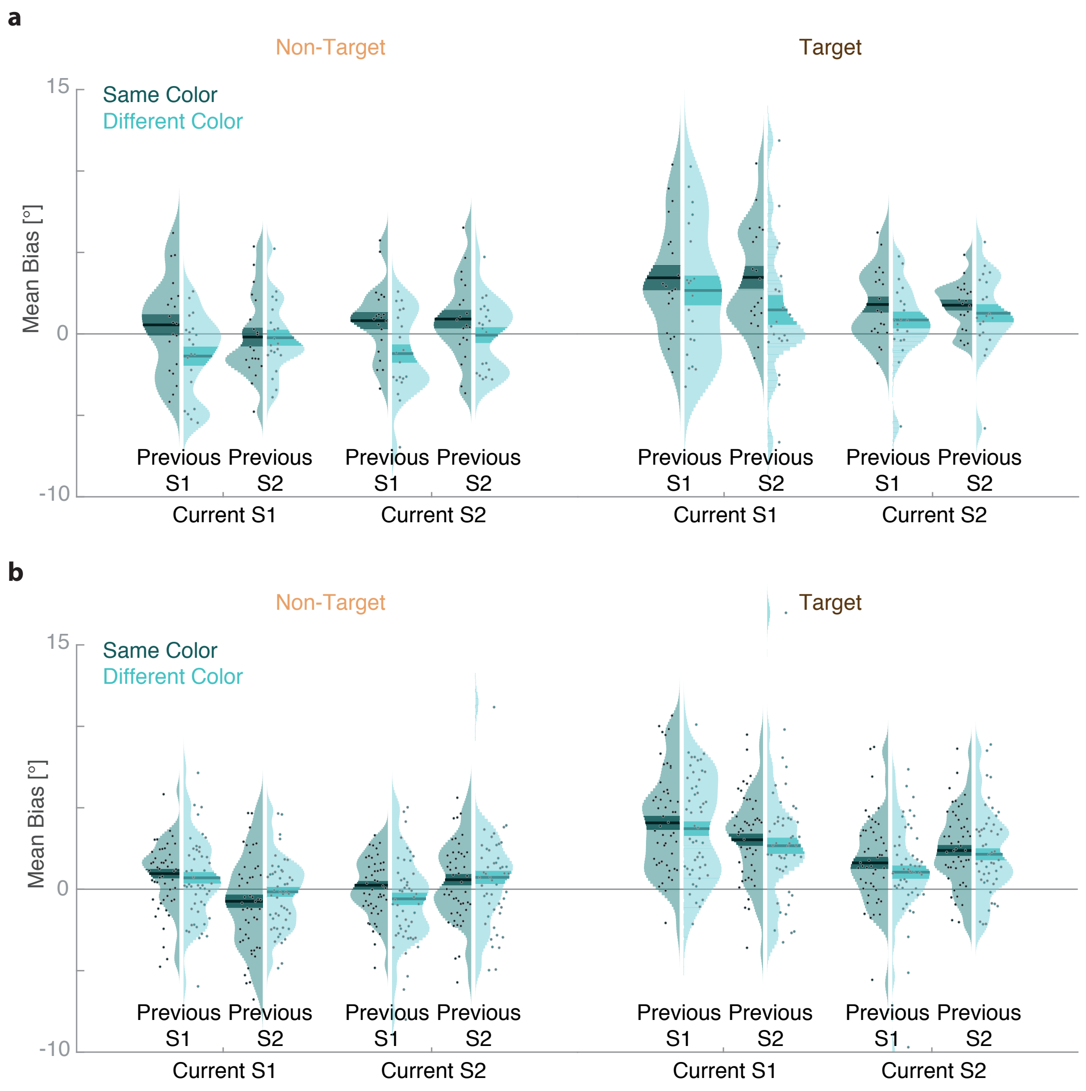

Supplementary Figure 2. Results for Experiments 1 and Experiment 2 with respect to whether the previous and the current item was presented as first or second stimulus in a trial (S1 or S2). In Experiments 1 (**a**) and Experiment 2 (**b**), the stimuli were presented sequentially. Previous studies observed that serial dependence is affected by the time that has passed between the previous and the current item, i.e. the closer in time two items are together, the stronger the serial dependence between them (Fischer & Whitney, 2014). Thus, in addition to our main analysis, we split the data with respect to whether the previous and the current item was S1 or S2. The figure depicts the resulting group mean response biases separately for each experimental condition. Please note that due to the reduction of trials per condition by splitting the data further, the mean bias was calculated using the same procedure as described for the interaction analysis (for details see Methods Experiment 1). The y-axis indicates the mean response bias in degrees with a positive bias indicating attraction toward a previous item. Solid lines represent the group mean with the surrounding opaque area indicating ± 1 standard error of the group mean (two experiments with independent samples; Exp. 1: n=20, Exp. 2: n=49) (see Supplementary Tables 5 and 6 for statistical results). The transparent areas reflect the distributions of the underlying individual mean biases. Colored circles represent the individual response biases. Source data are provided as a Source Data file.

Supplementary Table 5. Results for ANOVA analysis of Experiments 1 with respect to whether the previous and current item were S1 or S2.

Factor	*df*	*F*	*p-Value*	BF_{incl}	η_p^2
Color	1,19	15.120	**<.001**	**302.653**	0.443
Previous Item Position	1,19	0.198	.661	0.128	0.010
Current Item Position	1,19	1.483	.238	0.437	0.072
Previous Role	1,19	19.542	**<.001**	**$2.829*10^{10}$**	0.507
Color × Previous Item Position	1,19	1.213	.284	0.253	0.060
Color × Current Item Position	1,19	0.011	.917	0.168	0.001
Color × Previous Role	1,19	0.132	.721	0.188	0.007
Previous Item Position × Current Item Position	1,19	2.679	.118	0.287	0.124
Previous Item Position × Previous Role	1,19	1.953	.178	0.315	0.093
Current Item Position × Previous Role	1,19	6.399	**.020**	**14.910**	0.252
Color × Previous Item Position × Current Item Position	1,19	0.261	.615	0.232	0.014
Color × Previous Item Position × Previous Role	1,19	4.005	.060	0.797	0.174
Color × Current Item Position × Previous Role	1,19	2.447	.134	0.350	0.114
Previous Item Position × Current Item Position × Previous Role	1,19	0.125	.728	0.222	0.007
Color × Previous Item Position × Current Item Position × Previous Role	1,19	1.753	.201	0.508	0.084

Splitting the data with respect to whether the previous and the current item were S1 or S2 resulted in a 4-way ANOVA with the factors color (same/different), previous role (target/non-target), previous item position (previous S1/previous S2) and current item position (current S1/current S2). Please note that the factor "serial position" of our main analysis, which referred to whether the current and the previous item shared the same serial position or not (regardless of their actual position), was split here into two new factors: previous item position and current item position.
As in our main analysis, we found a significant effect of the task-relevant context feature color and of the previous role. Interestingly, the 4-way ANOVA showed that serial dependence was similar for current S1 and current S2 and was also similar when the previous item was actually S1 or S2. In addition, the ANOVA showed a significant interaction between current item position and previous role: the current S1 was more strongly attracted toward previous targets than toward previous non-targets as compared to the current S2.
The second-last column lists Bayes Factors (BF_{incl}) which indicate the contribution of a single factor or interaction. Bold accentuation indicates p-values < .05. Source data are provided as a Source Data file.

Supplementary Table 6. Results for ANOVA analysis of Experiments 2 with respect to whether the previous and current item were S1 or S2.

Factor	*df*	*F*	*p-Value*	BF_{incl}	η_p^2
Color	1,48	1.075	.305	0.190	0.022
Previous Item Position	1,48	0.789	.379	0.114	0.016
Current Item Position	1,48	12.893	**<.001**	**367.444**	0.212
Previous Role	1,48	114.310	**<.001**	**$5.586*10^{32}$**	0.704
Color × Previous Item Position	1,48	4.807	**.033**	**0.324**	0.091
Color × Current Item Position	1,48	0.808	.373	0.138	0.017
Color × Previous Role	1,48	0.718	.718	0.153	0.015
Previous Item Position × Current Item Position	1,48	28.762	**<.001**	**$8.227*10^{5}$**	0.375
Previous Item Position × Previous Role	1,48	0.302	.302	0.126	0.006
Current Item Position × Previous Role	1,48	16.744	**<.001**	**$2.882*10^{3}$**	0.259
Color × Previous Item Position × Current Item Position	1,48	1.392	.637	0.182	0.005
Color × Previous Item Position × Previous Role	1,48	1.229	.273	0.421	0.025
Color × Current Item Position × Previous Role	1,48	0.413	.523	0.194	0.009
Previous Item Position × Current Item Position × Previous Role	1,48	0.033	.857	0.151	0.001
Color × Previous Item Position × Current Item Position × Previous Role	1,48	0.024	.878	0.240	0.000

Splitting the data with respect to whether the previous and the current item were S1 or S2 resulted in a 4-way ANOVA with the factors color (same/different), previous role (target/non-target), previous item position (previous S1/previous S2) and current item position (current S1/current S2). For details, see Supplementary Table S1.

As in our main analysis, we found a significant effect of the task-relevant context feature serial position (revealed by the interaction between Previous Item Position × Current Item Position) and of the previous role. In addition, we also observed an effect of current item position, i.e. the current S1 showed a stronger attraction bias than the current S2. In contrast, serial dependence was similar for previous S1 and S2. We observed two additional interactions. First, there was a significant interaction between color and previous item position, i.e. color modulated serial dependence when the previous item was S1, but not when previous item was S2. While this interaction was significant, the Bayes Factor (BF_{incl}) indicated only inconclusive evidence. Second, we observed a significant interaction between the current item position and the previous role similar to Experiment 1, hinting at a stronger influence of the previous role on serial dependence for the current S1 than the current S2.

Taken together, the present additional analyses (Supplementary Tables S1 and S2) showed that the current S1 was more strongly impacted by previous targets than the current S2. This is in line with the finding that temporal proximity facilitates serial dependence (Fischer et al., 2014), as S1 was closer in time to the previous trial. As the current S1 was also maintained in working memory for a longer duration before report than S2, our results are also in accordance with the observation that serial dependence is increased by working memory (Bliss et al., 2017; Papadimitrou et al., 2015).

The second-last column lists Bayes Factors (BF_{incl}) which indicate the contribution of a single factor or interaction. Bold accentuation indicates p-values < .05. Source data are provided as a Source Data file.

Supplementary Note 1

The DoG fitting procedure initially introduced by Fischer and Whitney [1] incorporates both amplitude and width as separate parameters. However, it is possible that especially under higher noise levels their estimation is not independent from each other. To test whether amplitude and width estimates were interdependent in our data, we ran a simulation. We started with the effect of previous non-targets that just fell short of significance ($p = .0519$), observed in Experiment 1. To obtain realistic noise data, we subtracted the group-level serial dependence bias as fitted with the DoG method (amplitude: 0.71°, width: 0.1) from each subject's individual response error in the non-target condition of Experiment 1. Given this individual "baseline noise", a small amplitude effect as the one subtracted ($a = .71°$) is just at the border of significance. Higher noise levels will therefore not lead to significant results. For simulation, we reduced this baseline noise in five levels, from 100% of its original size to 20% in steps of 20% by multiplying the individual baseline noise with the reduction factor (e.g. with 0.8 for the 80% condition). On this noise, we individually added a serial dependence bias by adding a DoG function. We used three artificial biases with different amplitudes (the original ones from the contrast, .71° and 2.99° as well as the mean 1.85° of both) and a fixed w value of .065, which is the mean of the original w estimates of this contrast. To assess the effect the amplitude has on the estimation of the width parameter, we fitted the DoG model in each of these 5 (noise level) x 3 (amplitude) conditions and examined the relationship between amplitude and width (Supplementary Figure 3a).
We observed merely statistical trends for differences between the three amplitude conditions (low vs. high: $p = .072$; low vs. medium: $p = .075$; medium vs. high: $p =.087$; two-sided paired t-tests, Bonferroni corrected; see Supplementary Figure 3b). The different noise levels affected the width estimation mainly in the low-amplitude condition. Based on this finding we concluded that the amplitude might have a slight effect on the width estimation at a high noise level. However, when the amplitude was low, the variability of the width estimation was in a very small range (<1°). This range was far below any significant FWHM difference as observed in our four experiments (the lowest significant FWHM difference was 8.86°). This demonstrates that our results concerning the w parameter were not driven by amplitude differences.

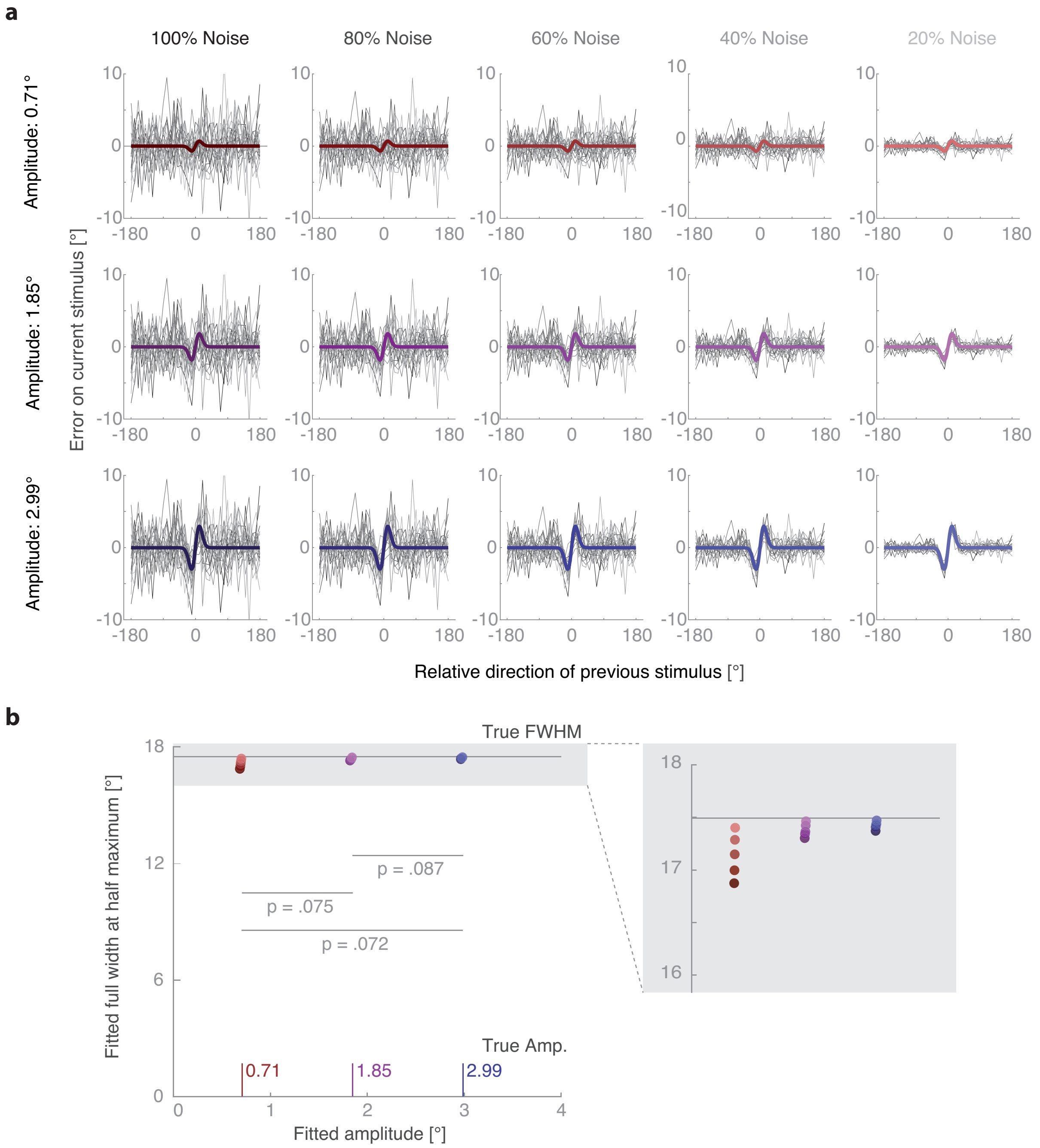

Supplementary Figure 3. Interdependence between amplitude and width estimates with the DoG fitting method under different noise levels. Panel **a**) shows the simulated data with their fittings. The individual mean response errors (ordinate; grey lines) are shown as a function of the motion direction difference (abscissa) between an item of the previous trial and the target of the current trial. Red, purple and blue lines depict the fitted DoG models, whereas red indicates the low amplitude condition, purple the medium amplitude condition and blue the high amplitude condition. **b**) The fitted parameters are depicted as colored dots, where the color indicates the amplitude of the artificial bias and the brightness the underlying noise level, from high (dark color) to low (light color). The x-axis indicates the estimated amplitude and the y-axis the estimated width expressed as full width at half maximum (FWHM). The grey shaded area shows a zoomed-in fraction of the y-axis to make the small differences visible. We observed *merely statistical trends* for differences between the three amplitude conditions (low vs. high: $p = .072$; low vs. medium: $p = .075$; medium vs. high: $p = .087$; two-sided paired t-tests, Bonferroni corrected). Source data are provided as a Source Data file.

Supplementary References

1. Fischer, J. & Whitney, D. Serial dependence in visual perception. Nat. Neurosci. 17, 738–743 (2014).
