# Supplementary material for: Context information supports serial dependence of multiple visual objects across memory episodes
Source: Nat Commun. 2020 Apr 22;11:1932. doi: 10.1038/s41467-020-15874-w (PMC7176712; doi:10.1038/s41467-020-15874-w)
Supplement: Supplementary file 3 — Reporting Summary [file 41467_2020_15874_MOESM3_ESM.pdf]

nature research

Corresponding author(s): PD Dr. Christoph Bledowski

Last updated by author(s): Mar 12, 2020

Reporting Summary

Nature Research wishes to improve the reproducibility of the work that we publish. This form provides structure for consistency and transparency in reporting. For further information on Nature Research policies, see Authors & Referees and the Editorial Policy Checklist.

Statistics

For all statistical analyses, confirm that the following items are present in the figure legend, table legend, main text, or Methods section.

n/a	Confirmed	
☐	☒	The exact sample size (*n*) for each experimental group/condition, given as a discrete number and unit of measurement
☐	☒	A statement on whether measurements were taken from distinct samples or whether the same sample was measured repeatedly
☐	☒	The statistical test(s) used AND whether they are one- or two-sided<br>*Only common tests should be described solely by name; describe more complex techniques in the Methods section.*
☒	☐	A description of all covariates tested
☐	☒	A description of any assumptions or corrections, such as tests of normality and adjustment for multiple comparisons
☐	☒	A full description of the statistical parameters including central tendency (e.g. means) or other basic estimates (e.g. regression coefficient) AND variation (e.g. standard deviation) or associated estimates of uncertainty (e.g. confidence intervals)
☐	☒	For null hypothesis testing, the test statistic (e.g. *F*, *t*, *r*) with confidence intervals, effect sizes, degrees of freedom and *P* value noted<br>*Give P values as exact values whenever suitable.*
☐	☒	For Bayesian analysis, information on the choice of priors and Markov chain Monte Carlo settings
☒	☐	For hierarchical and complex designs, identification of the appropriate level for tests and full reporting of outcomes
☐	☒	Estimates of effect sizes (e.g. Cohen's *d*, Pearson's *r*), indicating how they were calculated

Our web collection on statistics for biologists contains articles on many of the points above.

Software and code

Policy information about availability of computer code

Data collection	Matlab v 2012b (The MathWorks, Inc., Natick, Massachusetts, United States)<br>Psychtoolbox v 3.0.14 (Brainard, 1997; Pelli, 1997)
Data analysis	Custom analysis scripts are available via https://osf.io/azpwy/.<br><br>used software and toolboxes:<br>Matlab v 2018a (The MathWorks, Inc., Natick, Massachusetts, United States)<br>JASP v 0.11.1 (JASP Team, 2019)<br>Circular Statistics Toolbox v 1.21 (Berens, 2009)<br>EzyFit v 2.44 (Moisy, 2016)<br>BADS v 1.0.5 (Acerbi & Ma, 2017)<br>fLOESS v 2.1 (Marsh, 2016)<br>shadedErrorBar v 1.65 (Campbell, 2018)<br>Violin Plots for plotting multiple distributions (distributionPlot.m) v 1.15 (Jonas, 2017)

For manuscripts utilizing custom algorithms or software that are central to the research but not yet described in published literature, software must be made available to editors/reviewers. We strongly encourage code deposition in a community repository (e.g. GitHub). See the Nature Research guidelines for submitting code & software for further information.

Data

Policy information about availability of data

All manuscripts must include a data availability statement. This statement should provide the following information, where applicable:

- Accession codes, unique identifiers, or web links for publicly available datasets
- A list of figures that have associated raw data
- A description of any restrictions on data availability

Data are available via https://osf.io/azpwy.

Field-specific reporting

Please select the one below that is the best fit for your research. If you are not sure, read the appropriate sections before making your selection.

☐ Life sciences ☒ Behavioural & social sciences ☐ Ecological, evolutionary & environmental sciences

For a reference copy of the document with all sections, see nature.com/documents/nr-reporting-summary-flat.pdf

Behavioural & social sciences study design

All studies must disclose on these points even when the disclosure is negative.

Study description	We conducted a quantitative behavioral study to investigate the role of context features for serial dependence in situations when multiple visual objects have to be memorized in a memory episode.
Research sample	In Experiment 1, 22 participants were recruited and 20 completed the experiment (10 male, age range: 18-32 years, age mean: 23.3 years). In Experiment 2, 51 participants completed the experiment and 49 of them were included in the final analysis (19 male, age range: 18-33 years, age mean: 23.8 years). Two participants were excluded from the final analysis due to poor task performance (SD of report error > 3 SDs of the sample mean). In Experiment 3, 21 participants completed the experiment and 20 of them were included in the final analysis (4 male, age range: 19-30 years, age mean: 22.9 years). One participant was excluded from the final analysis due to poor task performance (SD of report error > 3 SDs of the sample mean). In Experiment 4, 20 participants completed the experiment (7 male, age range: 19-29 years, age mean: 23.15 years). The sample sizes for Experiments 1, 3 and 4 (n=20) were based on previous similar experiments (Czoschke et al., 2019: n=15 and n=19; Fritsche & DeLange 2017: n=25). The sample size in Experiment 2 was increased to obtain more conclusive results about one effect that was close to significance in Experiment 1 (p = .0519). We aimed at doubling our sample in comparison to Fritsche & DeLange (n=25), which resulted in a final sample of n=49. No participant took part in more than one experiment of the study. All participants had normal or corrected-to-normal vision. The participants were students at the Goethe-University Frankfurt or the Fresenius University of Applied Sciences Frankfurt.
Sampling strategy	We used a within-subject design with random participant sampling in both experiments. The sample size was based on previous studies on serial dependence (see above: Research samples). In our previous study (Czoschke et al., 2019), we could reliably observe serial dependence with a very similar paradigm. In comparison to this study, the number of experimental trials was doubled in order to have the same number of trials per condition for our comparisons (e.g. same vs. different color). As Experiments 1, 3 and 4 have similar participant numbers and Experiment 2 a crucially increased participant number in comparison to Czoschke et al. (2019), the tested sample sizes are sufficient to compare serial dependence between two conditions.
Data collection	The behavioral responses were collected and stored on a computer. Up to three participants completed the experiment in parallel in a behavioral lab room, visually and acoustically shielded from one another. Researchers were not blind with regard to experimental condition or study hypothesis.
Timing	Experiments 1 and 2: August 2017 to February 2018; Experiments 3 and 4: October to November 2019
Data exclusions	In Experiment 2 two participants and in Experiment 3 one participant were excluded from the final analysis due to poor task performance (SD of report error > 3 SDs of the sample mean).
Non-participation	Two participants dropped out of Experiment 1 before starting the actual experiment because they had problems with the behavioral task during the practice phase.
Randomization	Participants were not allocated into experimental groups as both experiments had a within-subjects design.

Reporting for specific materials, systems and methods

We require information from authors about some types of materials, experimental systems and methods used in many studies. Here, indicate whether each material, system or method listed is relevant to your study. If you are not sure if a list item applies to your research, read the appropriate section before selecting a response.

Materials & experimental systems

n/a	Involved in the study	
☒	☐	Antibodies
☒	☐	Eukaryotic cell lines
☒	☐	Palaeontology
☒	☐	Animals and other organisms
☐	☒	Human research participants
☒	☐	Clinical data

Methods

n/a	Involved in the study	
☒	☐	ChIP-seq
☒	☐	Flow cytometry
☒	☐	MRI-based neuroimaging

Human research participants

Policy information about studies involving human research participants

Population characteristics	See above.
Recruitment	Participants were recruited from an internal participant database and via online advertisements in university specific facebook groups. Therefore, only students participated in our study and received a small monetary reimbursement or study credit, which is common in cognitive psychology research. We do not expect our results to be impacted by the non-representative sample since the study investigated basic perception and memory processes.
Ethics oversight	The study was approved by the Ethics Committee of the Medical Faculty of the Goethe-University Frankfurt am Main

Note that full information on the approval of the study protocol must also be provided in the manuscript.
